# Augmented Reality for Guideline Presentation in Medicine: Randomized Crossover Simulation Trial for Technically Assisted Decision-making

**DOI:** 10.2196/17472

**Published:** 2021-10-18

**Authors:** Andreas Follmann, Alexander Ruhl, Michael Gösch, Marc Felzen, Rolf Rossaint, Michael Czaplik

**Affiliations:** 1 Department of Anesthesiology Faculty of Medicine Rheinisch-Westfälische Technische Hochschule Aachen University Aachen Germany; 2 Tech2go Mobile Systems GmbH Hamburg Germany; 3 Medical Direction Emergency Medical Service City of Aachen Aachen Germany

**Keywords:** augmented reality, smart glasses, wearables, guideline presentation, decision support, triage

## Abstract

**Background:**

Guidelines provide instructions for diagnostics and therapy in modern medicine. Various mobile devices are used to represent the potential complex decision trees. An example of time-critical decisions is triage in case of a mass casualty incident.

**Objective:**

In this randomized controlled crossover study, the potential of augmented reality for guideline presentation was evaluated and compared with the guideline presentation provided in a tablet PC as a conventional device.

**Methods:**

A specific Android app was designed for use with smart glasses and a tablet PC for the presentation of a triage algorithm as an example for a complex guideline. Forty volunteers simulated a triage based on 30 fictional patient descriptions, each with technical support from smart glasses and a tablet PC in a crossover trial design. The time to come to a decision and the accuracy were recorded and compared between both devices.

**Results:**

A total of 2400 assessments were performed by the 40 volunteers. A significantly faster time to triage was achieved in total with the tablet PC (median 12.8 seconds, IQR 9.4-17.7; 95% CI 14.1-14.9) compared to that to triage with smart glasses (median 17.5 seconds, IQR 13.2-22.8, 95% CI 18.4-19.2; *P*=.001). Considering the difference in the triage time between both devices, the additional time needed with the smart glasses could be reduced significantly in the course of assessments (21.5 seconds, IQR 16.5-27.3, 95% CI 21.6-23.2) in the first run, 17.4 seconds (IQR 13-22.4, 95% CI 17.6-18.9) in the second run, and 14.9 seconds (IQR 11.7-18.6, 95% CI 15.2-16.3) in the third run *(P*=.001). With regard to the accuracy of the guideline decisions, there was no significant difference between both the devices.

**Conclusions:**

The presentation of a guideline on a tablet PC as well as through augmented reality achieved good results. The implementation with smart glasses took more time owing to their more complex operating concept but could be accelerated in the course of the study after adaptation. Especially in a non–time-critical working area where hands-free interfaces are useful, a guideline presentation with augmented reality can be of great use during clinical management.

## Introduction

Guidelines as well as standard operating procedures consist of recommendations for action in corresponding diseases or injuries and they contribute to a standardized, high-quality patient care, preferably independent from the user’s experiences [1]. Many studies have certainly proven the benefit of guidelines but have also shown their deficiencies in appropriate implementation. Most of these decision trees are complex and are often misapplied or not applied at all [2,3]. Other studies have shown that the simultaneous visual presentation of the guidelines as well as electronic decision-support systems have improved their implementation [4,5]. Various guidelines are therefore available as printed versions or posters in clinics.

Electronic tools such as the display on tablet computers are beneficial [6]. A display using augmented reality in smart glasses has the decisive advantage of a hands-free concept, which enables operation of the smart glasses and the simultaneous use of hands as important tools for examination or treatment. Moreover, these smart glasses can collect and save all the guidelines to use case-related data iteratively [7]. Smart glasses have already been applied in experimental implementations, especially in the field of telesupervision for intraoperative teleconsultation from a surgeon’s point of view [8] or to transfer medical knowledge from medically highly advanced countries to low-income countries [9]. Smart glasses can display patient-related information such as radiographic images [10] as well as the name of the currently consulted patient and his/her vital parameters [11].

In emergency and disaster medicine, decisions are taken in a short amount of time. However, the rapid expansion of medical literature has led to a high publication rate of various guidelines, further limiting the rapid implementation into practice [12,13]. The introduction of electronic-based decision-making systems could provide a decisive advantage in the use of clinical guidelines. This advantage is additionally strengthened by the fact that emergency medicine has been an early adaptor for a variety of technology-based tools [14,15]. Carenzo et al [16] tested the Google glasses for the first time in a large-scale emergency with 100 theoretical emergency patients and used a triage algorithm that was implemented into an app, and they reported that smart glasses have a high potential to be used as decision-support systems.

To provide iterative technical support for triage in case of mass casualty incidents and as an example for any other guideline-related decision, we have developed a guideline presentation as a decision support running on smart glasses (Recon Jet, Recon Instruments). Guidelines were displayed on a small monitor on the smart glasses. With simple operating gestures performed on an optical touchpad, menu items can be selected. Although the results showed a clearly improved implementation of the guideline, they also showed that more time was necessary for the processing of all decisions [17]. This finding suggests that a lot of time is needlessly wasted due to the unusual application of the smart glasses’ operating concept. Only after a long-term application, the user achieves a learning effect, which accelerates the app performance. The use of a device with the usual display and choice of decisions such as a tablet computer could save a lot of time. However, these devices are not appropriate for use in emergency cases: hands cannot be used freely, body fluids can come into contact with the devices compared to smart glasses because of the short distance between the device and patient, and thereby cause further transmission of germs.

The objective of this study was to examine if smart glasses are in an inferior position compared to tablet computers in the case of guideline presentation. This study will evaluate if the application of a guideline displayed by smart glasses is performed much slower than that performed using a tablet computer and if the time required can be reduced by the learning effect after longer use. Another purpose of this study was to analyze if the test person’s experience in emergency medicine has an influence on the time and accuracy of the triage. Especially in case of a mass casualty incident with shortage of staff, it is important to transfer medical staff from other departments with less experience in emergency medicine to the place of accident to support the experienced staff. Therefore, this study analyzes if staff with no or less experience in emergency or disaster medicine can also triage patients quickly and accurately.

## Methods

### Study Design and Triage

**Figure 1:**
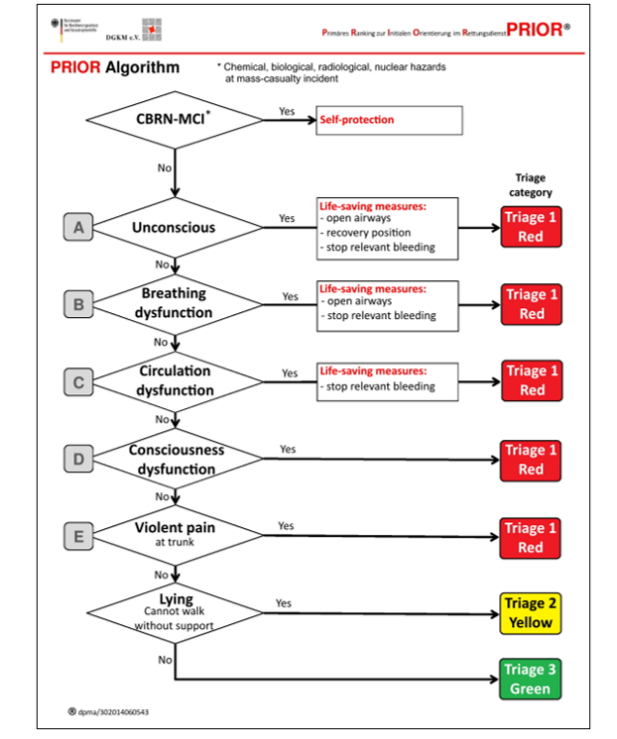
English translation of the algorithm for PRIOR (primary ranking for initial orientation in rescue service) triage [18].

This study is a randomized controlled crossover simulation trial for guideline presentation. A so-called “PRIOR” (primary ranking for initial orientation in rescue service) triage algorithm [18] was applied and displayed on a tablet PC or smart glasses (Figure 1). By using the triage algorithm and following its flowchart, the test person’s task was to determine 1 of the 3 triage categories for each of the 30 theoretical patient cases.

First, the user of the PRIOR algorithm has to decide if there are any chemical, biological, radiological, or nuclear hazards concerning the injured person that should be triaged. If there are any of these hazards, the triaging person is instructed to set the focus on self-protection and not to triage the injured patient. This concept should prevent the triaging person from spreading the contamination. If these hazards do not exist, the test subject can triage the injured person by following the further paths of the algorithm. The main structure of the algorithm is based on the ABCDE-scheme (airway, breathing, circulation, disability, environment) that is applied in emergency medicine.

The user has to decide if the current criterion (eg, unconscious) can be applied concerning the injury of the triaged patient and choose between “Yes” or “No.” At the end of each path, the algorithm announces 1 of the 3 possible triage categories (red, yellow, or green), and the triage of 1 injured patient is finished. The 3 triage categories represent the urgency of a fast treatment of the injured person. An injured person that is triaged red (emergency) should be treated immediately; yellow (urgent) and green (routine) triaged persons should be treated within 1 hour and 4 hours, respectively. [16].

### Electronic Devices

The following 2 devices for technical support were used by the test subjects to triage 30 theoretical emergency patients after receiving approval from the local ethics committee (Aachen, Germany; EK320/16):

1. Tablet PC as an established control device (Samsung Galaxy Tab A 2016, Samsung Electronics AG)
2. Smart glasses as a new technical method of support (Recon Jet, Recon Instruments Corp).

The tablet PC was operated using a touchscreen, whereas the smart glasses were operated using several buttons (touchpad, switch with 2 buttons). The front switch is used to confirm a choice, while the back switch is used as the return key. Moreover, with the visual touchpad that is located at the right-hand outer side of the smart glasses, the marked choice can be changed by swiping with the index finger to the left or right.

### Test Procedure

**Figure 2:**
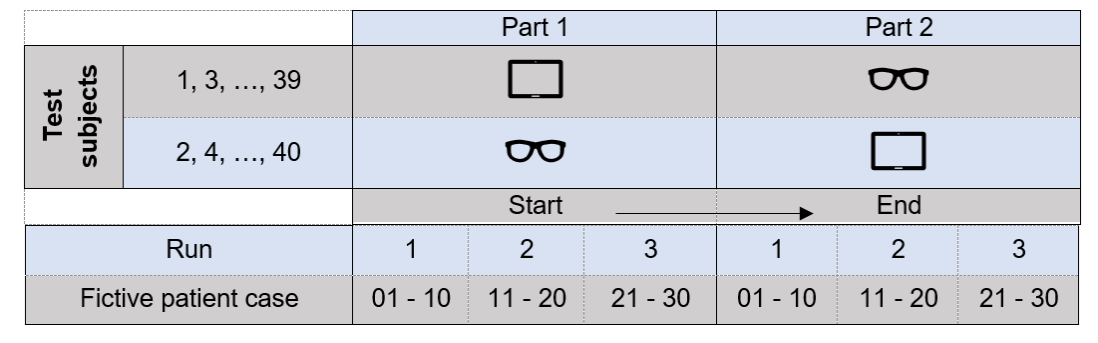
The test procedure in the crossover design with different devices: tablet PC (as a usual device for control group) and smart glasses.

The triage time and the correct application of the guideline (“PRIOR”) via smart glasses were analyzed and compared with the already established operating concepts of the tablet. Forty volunteer test subjects were included in this study by using the triage as an exemplary application of a guideline with the help of theoretical case descriptions. During the introduction, the test subjects were allowed to try out the relevant operating buttons on the smart glasses. Depending on the test subject number, which was randomly assigned, participants started either with the tablet (uneven subject number) or with the smart glasses (even subject number). First, the triage of 30 fictive patients was performed with either only the smart glasses (n=20) or only the tablet (n=20). After that, the test subjects performed the triage on the same patients in the identical order with the other electronic device (Figure 2). Moreover, after each run of 10 patient triages, a break of 1 minute was taken.

During the trial, the test subjects only used the smart glasses or the tablet with the installed app, whereas the presentation of the cases, the monitoring of time, and the documentation of the correct use concerning the applied guideline was performed by the investigator. The fictive cases were presented on the screen of a desktop PC. The attended time of the guideline’s application was defined as the primary outcome parameter, and the correct application was defined as the secondary outcome. The start of the time measurement was defined as the beginning of the presentation of the fictive patient and the end was defined by determining the triage category.

### Fictive Patients in This Study

In this study, the cases designed for triage with technical support consisted of 30 different theoretical patients separated into 3 runs with 10 fictive patients each. The cases were text-based scenarios, and the text of each case was presented on a display of a desktop computer and the test persons had the task of reading the text of the case and to triage the patient that was described in the text. The description of the patients involved typical injuries that can be expected at a mass casualty incident, for example, the first run consisted of 10 patients with injuries because of a multi-vehicle accident. Each run contained the same triage results following the same paths in the decision tree for this guideline.

A comparison of all 3 runs (complexity of cases, triage categories, quantity of signs) for the subsequent evaluation was included to compare the triage results within the study process and between the devices. Different linguistic designs of comparable cases were used to mask the similarities preventing the recognition of comparable patients. The description of the cases consisted of short sentences, and no medical terms were used so that medical novices as well as medical experts could understand the description of the cases. In the following sentences, 3 exemplary descriptions of the theoretical patients that were applied in the study are listed with the triage category in brackets:

1. At a traffic collision, an involved person runs in your direction. He speaks normally and seems to be fully orientated (triage category: green).
2. The codriver of a passenger car is trapped in his car too. He cannot move his legs but he is awake and approachable. He breathes normally and has only little pain (triage category: yellow).
3. There is a trapped person under a seat of a bus. The person’s breaths are very flat and very fast, but she is orientated (triage category: red).

### Volunteers in This Study

The volunteers took part in this study in November and December 2017. They were recruited by an information letter with information about the workflow and intention of the study. The information letter was presented on a board where all public studies of the University Hospital of the RWTH (Rheinisch-Westfälische Technische Hochschule) Aachen are advertised. The subjects who were interested in participating in this study had to email or call the director of the study if they had any questions regarding the study or had the intention to take part in the study.

The participation in this study was voluntary and the participating students had no advantages or disadvantages by participating in this study along with their university studies. The only exclusion criterion of this study was the inability to use smart glasses because of visual impairment (inability to see the information on the display of the smart glasses or tablet screen clearly despite wearing glasses or contact lenses). Only 1 of the 41 test subjects was excluded because of visual impairment. Prior medical knowledge was not required because the used ”PRIOR” triage algorithm can be applied by nonmedical subjects. Moreover, special technical knowledge or the application of technical devices in the past was also not an inclusion criterion. The test persons received a financial incentive of €10 (US $11.73).

### Android App

**Figure 3:**
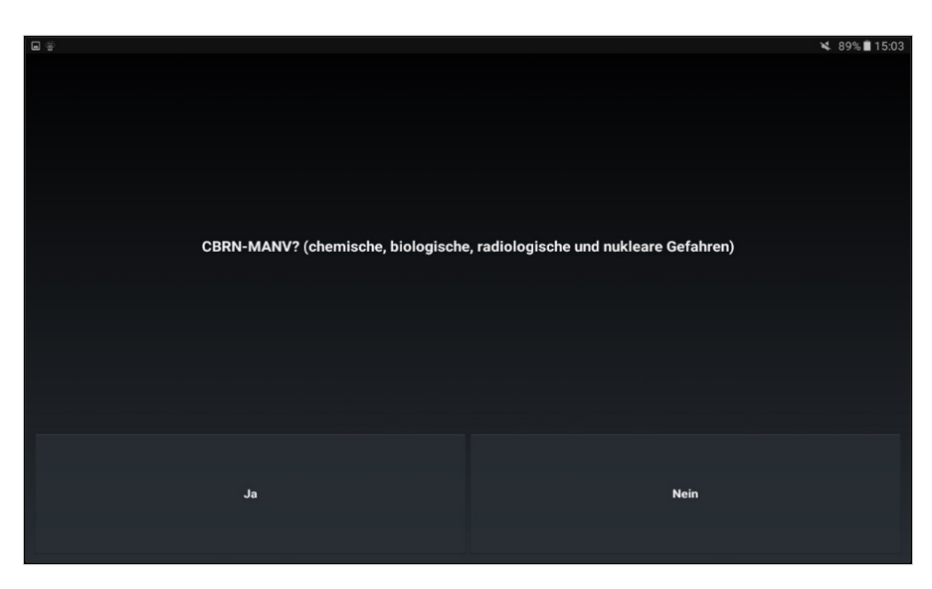
First question of the PRIOR (primary ranking for initial orientation in rescue service) triage algorithm on the user interface of the app on a tablet PC. The selection is done via a touch on the screen; answers are Yes or No. CBRN: chemical, biological, radiological, nuclear; MANV: mass casualty incident.

**Figure 4:**
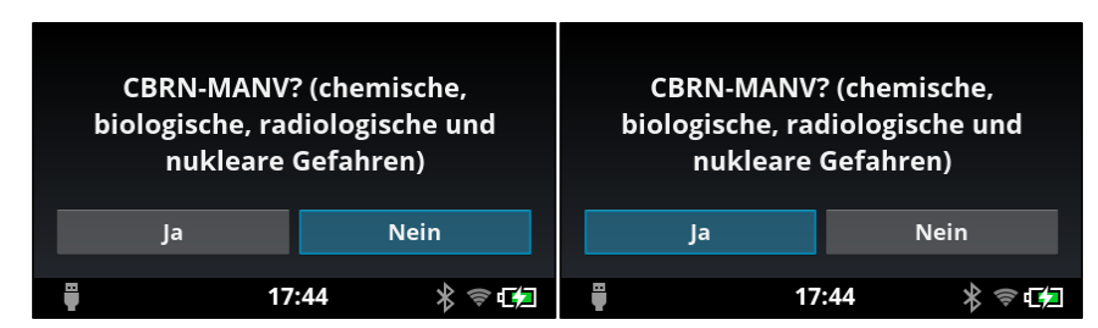
User interface of the app on the smart glasses. The answer (Yes or No) is chosen on an optical touchpad and confirmed with the confirmation key. CBRN: chemical, biological, radiological, nuclear; MANV: mass casualty incident.

In the Android app “AUDIME” (Augmented Disaster Medicine, Tech2go Mobile Systems GmbH), which was designed for application in smart glasses as well as on the tablet PC, individual decision criteria of the guidelines are displayed to support test subjects with the triage of the respective case. The test subject’s task was to decide whether the respective criterion of the algorithm can be applied on the patient and to choose accordingly between “Yes” or “No.” The app was programmed in a way that the next criterion is displayed only after the previous criterion has been answered. This stepwise processing of the exact decision tree was necessary to avoid skipping of questions. Regarding the operating of the “AUDIME” app, there were device-specific differences: with the tablet PC, questions of the algorithm were answered by one-time typing on the Yes button or No button on the tablet display (Figure 3). Because of the smart glasses’ different operating concept, answers with reference to the questions of the algorithm were marked as “Yes” or “No” on the response fields. The marked answer could be changed by a one-time swipe on the touchpad of the smart glasses and chosen by a press on the confirmation key (Figure 4).

### Statistical Analysis

Data analysis was performed with a nonparametric distribution of the primary outcome parameters using the Mann-Whitney *U* test and the Wilcoxon test for independent samples (*P*=.05). SPSS Statistics 23.0.0.2 (IBM Corp) was used for statistical evaluation. All data included median or arithmetic mean, IQR, and 95% CI.

## Results

### Sample Characteristics

A total of 40 test subjects took part in this trial: 53% (21/40) were males and 48% (19/40) were females. The age of the test subjects ranged between 18 and 37 years, with a median age of 24 years (Table 1). Approximately 85% (34/40) of all the test subjects had medical knowledge because of their medical studies or a medical job. The proportion of students among all the participants was 93% (37/40); 29 volunteers were students of human medicine and 4 were students of dentistry. With regard to experience in emergency medicine, the subjects had the following experience: none (n=25), 1-100 hours (n=9), 101-1000 hours (n=5), and >1000 hours (n=1). None of the volunteers had experience with the use of smart glasses or the “AUDIME” Android app.

**Table 1:** Demographic data of the volunteers, depending on the device used in part 1.

Demographics	Tablet PC (n=20)	Smart glasses (n=20)
Females (n)	9	10
Males (n)	11	10
Age (years), median (IQR)	23.5 (19-37)	24.0 (18-35)

**Figure 5:**
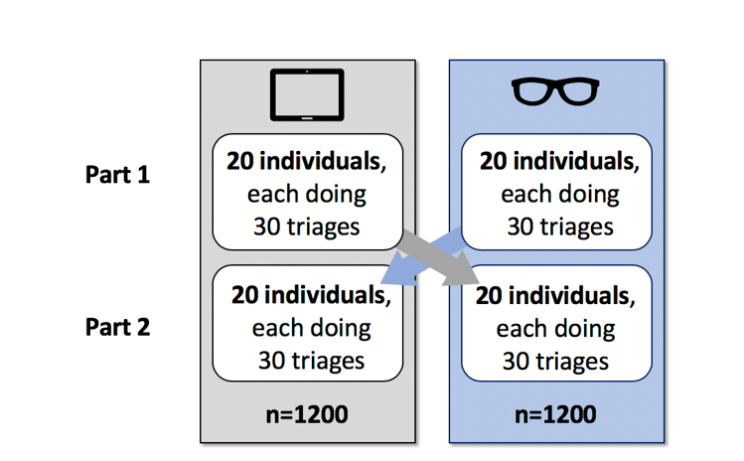
Number of triages per device group: tablet PC (as a usual device for control group) and smart glasses.

All 40 test subjects examined 30 fictive patients and applied the integrated guideline in each case by using both technical devices in a crossover, which resulted in 2400 triages: 1200 with the support of the smart glasses and 1200 with the support of the tablet PC (Figure 5).

### Duration of Triage

The median time to triage with smart glasses was 17.5 seconds (IQR 13.2-22.8, 95% CI 18.4-19.2) and that to triage with the tablet was 12.8 seconds (IQR 9.4-17.7, 95% CI 14.1-14.9). Thirty triages were subdivided into 3 sequential runs with 10 triages each. All 30 triages were finished with 1 device and subsequently with the other electronic device. The triage time within the same run with the tablet was significantly faster than that with the smart glasses for all 3 runs, which means that in total, triage using the tablet was significantly faster (Wilcoxon test, run 1: *z*=–10.178, *P*<.001; run 2: *z*=–10.373, *P*<.001; run 3: *z*=–10.697, *P*<.001; in total: *z*=–17.898, *P*<.001).

With the smart glasses, the median triage time was 21.5 seconds (IQR 16.5-27.3, 95% CI 21.6-23.2) in the first run, 17.4 seconds (IQR 13-22.4, 95% CI 17.6-18.9) in the second run, and 14.9 seconds (IQR 11.7-18.6, 95% CI 15.2-16.3) in the third run, whereas with the tablet, triage in the first, second, and third run took 15.0 seconds (IQR 11.0-20.8, 95% CI 16.1-17.7), 12.8 seconds (IQR 9.4-17.0, 95% CI 13.5-14.9), and 11.0 seconds (IQR 8.6-15.4, 95% CI 11.8-12.9), respectively.

**Figure 6:**
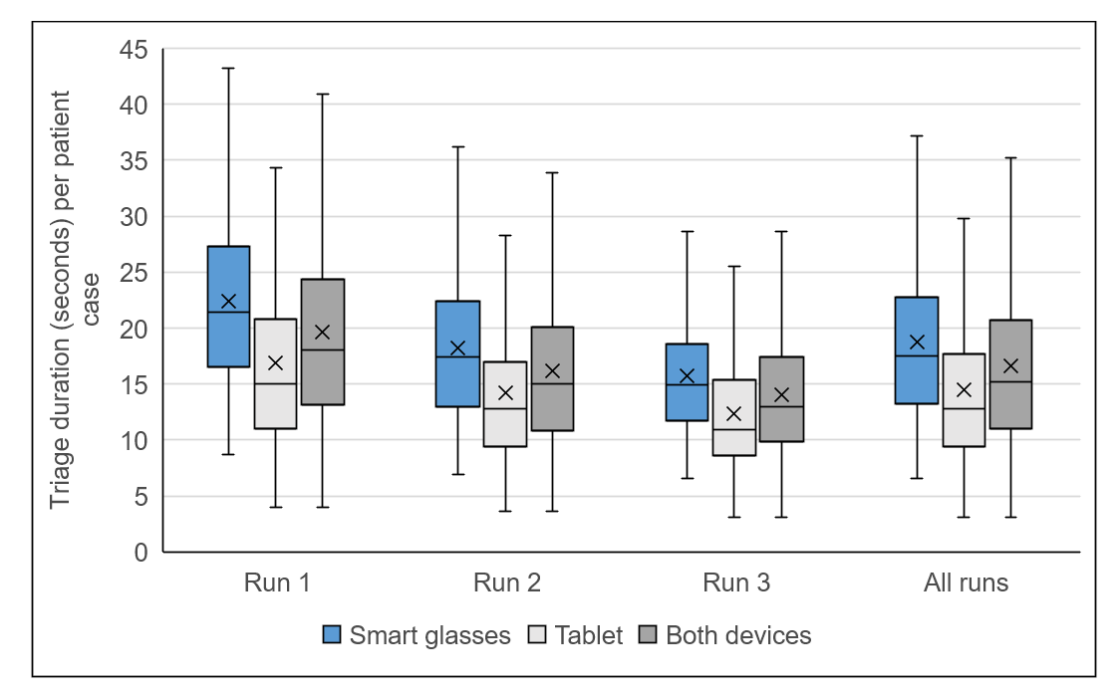
Box plot of triage duration (in seconds) per case over all trial parts depending on device and run.

Both with the smart glasses and the tablet, significantly faster triage time was achieved in the subsequent runs compared to the triage time in the previous run (Wilcoxon test, run 1 vs 2 with smart glasses: *z*=–9.332, *P*<.001; run 2 vs 3 with smart glasses: *z*=–7.169, *P*<.001; run 1 vs 2 with tablet: *z*=–7.582, *P*<.001; run 2 vs 3 with tablet: *z*=–5.191, *P*<.001). Moreover, the scattering of the triage times in consideration of the interquartile range was less for both devices in the subsequent runs compared with that in the previous runs (Figure 6).

**Figure 7:**
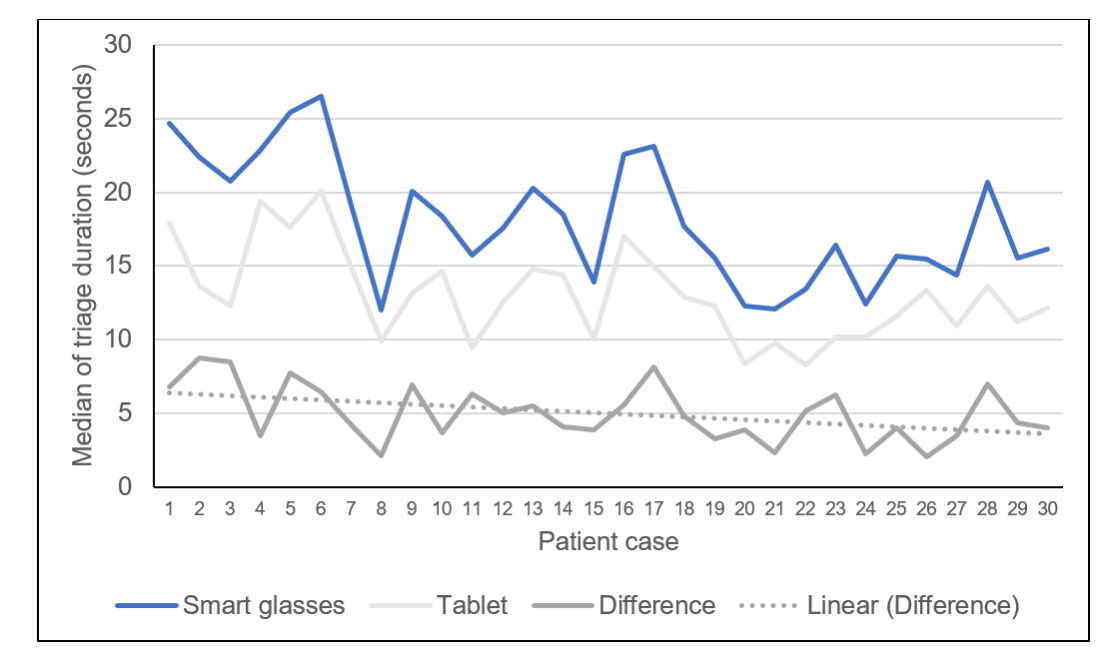
Line diagram showing the medians of triage duration (in seconds) depending on device and case as well as the calculated time difference between both devices.

With regard to the triage time for all the test subjects, the median triage time concerning all 30 patients with the tablet was lesser than that with the smart glasses (Figure 7). However, the time difference between both electronic devices decreased as the study progressed (with the increasing number of cases). Considering the respective medians, triage with the smart glasses took 5 seconds (IQR –0.9 to 11.8, 95% CI 4.5-6.5) in the first run, 4.1 seconds (IQR –0.3 to 8.5, 95% CI 3.3-4.8) in the second run, and only 3.5 seconds (IQR –0.2 to 7.0, 95% CI 2.8-4.0) longer than that with the tablet in the last run. Here, considering the difference in the triage time between both devices, the additional time needed with the smart glasses could be reduced significantly comparing the subsequent runs to its previous run (Wilcoxon test: run 1 vs 2, *z*=–3.142, *P*=.002; run 1 vs 3: *z*=–4.805, *P*<.001; run 2 vs 3: *z*=–2.181, *P*=.03).

The first triage of all 30 fictive patients (before changing the device) with the used device showed a median time of 17.7 seconds (IQR 13.5-23.1, 95% CI 18.6-19.5), whereas the retriage (after device change) with the other device required 12.6 seconds (IQR 9.5-17.4, 95% CI 13.8-14.6) and was therefore significantly faster (Wilcoxon test: *z*=–20.090, *P*<.001). Similar results appeared comparing the triage time of the same device depending on the first or second application for triage by the test subject. That way, triage with smart glasses took about 3.5 seconds and triage with the tablet took almost 6 seconds longer compared to those test subjects who started their triage with this device with those test subjects who used this device secondly (Mann-Whitney *U* test, smart glasses: *z*=–8.696, *P*<.001; tablet: *z*=–16.372, *P*<.001). Thus, triage with smart glasses took the longest time in the case of the triage started with the smart glasses (median 19.1 seconds, IQR 15.3-24.7, 95% CI 19.8-21.0). Triage using the tablet was faster after having used the smart glasses (median 10.2 seconds, IQR 8.0-13.6, 95% CI 10.9-11.6). Moreover, test persons with experience in emergency medicine (n=15) needed a median time of 14.5 seconds (IQR 10.3-19.5, 95% CI 15.1-16.0) to triage, whereas inexperienced volunteers (n=25) were significantly slower (Mann-Whitney *U* test: *z*=–5.018, *P*<.001) with a median of 15.7 seconds (IQR 11.3-21.5, 95% CI 16.9-17.7).

### Accuracy of the Devices

Approximately 86.3% (95% CI 0.84-0.88) of all triages were finished correctly by using smart glasses and 85.6% (95% CI 0.84-0.88) of all triages were finished correctly with the application of the tablet (Table 2). Within the same run as well as in total, there was no significant difference between both devices and their triage reliability (Wilcoxon test: run 1, *z*=–0.452, *P*=.65; run 2, *z*=–0.000, *P*>.99; run 3, *z*=–0.696, *P*=.46; in total, *z*=–0.727, *P*=.47). The triage accuracy with both devices during the trial showed the following values: 85% accuracy in the first run (95% CI 0.83-0.88), 82% accuracy in the second run (95% CI 0.80-0.85), and 90% accuracy in the third run (95% CI 0.88-0.92).

**Table 2:** Triage accuracy with the used device and runs.

Runs	Accuracy with smart glasses (%), mean (95% CI)	Accuracy with tablet PC (%), mean (95% CI)
Run 1	85.0 (81.5-88.5)	85.8 (82.3-89.2)
Run 2	82.3 (78.5-86.0)	82.3 (78.5-86.0)
Run 3	89.5 (86.5-92.5)	90.8 (87.9-93.6)
Total	86.3 (84.3-88.2)	85.6 (83.6-87.6)

Consequently, triage reliability was the highest in the last run. Furthermore, test subjects using smart glasses had a significantly more reliable triage in the third run than that in the first 2 runs (Wilcoxon test: run 1 vs 3, *z*=2.025, *P*=.04; run 2 vs 3, *z*=3.558, *P*<.001) and were significantly more precise with the tablet in the third run than that in the second run (Wilcoxon test: run 2 vs 3, *z*=–3.057, *P*=.002). Test persons with experience in emergency medicine (n=15) performed with an average accuracy of 91.6% (95% CI 0.90-0.93), whereas the inexperienced volunteers (n=25) triaged significantly less accurately (Mann-Whitney *U* test: *z*=–6.200, *P*<.001) with an accuracy of 82.5% (95% CI 0.81-0.84).

## Discussion

### Principal Results

In this study, a significantly faster triage time was achieved with the tablet compared to that with the smart glasses in all 3 runs as well as in total. While triage with the tablet took a median of 12.8 seconds, that with the smart glasses took a median of 17.5 seconds. Consequently, triage with the tablet was approximately 27% faster. Repetitive usage led to a learning curve with steadily decreasing time consumption. The accuracy of the triages was not different between using smart glasses or the tablet.

The differences between using both the devices can be attributed to the fact that the different operating concepts of both the devices have an enormous influence on the triage time and thus show great potential in the use of clinical guidelines. Therefore, future developments of these electronic devices should place a special focus on an easier operating concept to enable faster decision-making. A comparison of the triage time of smart glasses with triage times in other studies is not possible, although there have been studies with smart glasses in use without recordings of the triage times [16,19].

During this study, test subjects using both devices achieved an increasingly faster triage time as they became familiar with the guidelines and thus, the triage of patients. In addition, the use of smart glasses needed the subjects to adapt to an unfamiliar operating system, which later led to a constant reduction in the time difference between using smart glasses and tablet in numerous triages. In contrast, an adaptation to the tablet’s operation could be neglected owing to an intuitive operation. Taking the respective medians into consideration, triage with smart glasses took 6.5 seconds longer in the first run, 4.7 seconds in the second run, and 4.0 seconds longer in the third run compared to triage with the tablet. However, the time difference between both the devices could be reduced in the following runs as the test subjects became familiar with the operation of the smart glasses and finished triages faster.

The triage result using the guideline with smart glasses was correct in 86.3% of the fictive cases and that with the tablet was correct in 85.6% of the cases. Thus, the operation concepts of the devices within the same test conditions did not have a significant impact on the triage accuracy. In other triage studies with electronic triage support (ie, personal digital assistant, tablets, or computer), a comparable triage accuracy of 79%-83% has been achieved. However, only 1 electronic device was used in these studies, and therefore, the effect of the operation concept on the reliability of triage could not be differentiated [20,21].

Volunteers with experience in emergency medicine achieved significantly (*P*<.001) faster and more reliable triage results (median 14.5 seconds, 91.6% accuracy) compared to the inexperienced test subjects (median 15.7 seconds, 82.5% accuracy). Therefore, this study showed that the time to triage and the accuracy depends on the experience in emergency medicine of the triaging person. Nevertheless, our study shows that subjects inexperienced in emergency medicine knowledge are also able to triage at a mass casualty incident by using electronic devices and the PRIOR triage algorithm because they also achieved fast and accurate triage results. Owing to the good triage results of the test subjects who did not have experience in disaster or emergency medicine, it was not a methodical flaw that these volunteers took part in the study, and experience in emergency medicine was not defined as an inclusion criterion in this study.

This study shows the exemplary display of standardized treatment cords in augmented reality and the related information transfer. Thus, this study proves that standardized algorithms can be displayed with the help of electronic devices and therefore enable users to have direct access to compressed and situation-related information. This is especially relevant for medical care as well as other departments in which standardized guidelines exist and simultaneously targeted effective action is of crucial importance. The use of electronic-based decision-making systems such as smart glasses can help in this regard to quickly overcome the initial alienation and complexity of many clinical guidelines and enable an effective implementation in clinical patient care. In addition, it has been shown that by using electronic support, even inexperienced persons become capable of gradually making decisions within these guidelines, which makes the use of these devices also suitable for education purposes.

The operating concept of smart glasses is still unfamiliar compared to the simple touchscreen on tablet computers and smartphones. Therefore, it is recommended that paramedics use smart glasses and the triage algorithm regularly to triage emergency patients to receive frequent practice with the use of the triage algorithm and electronic device. For this reason, the implementation of further guidelines and algorithms that are useful for paramedics in all daily emergency medicine is preferable. In addition, paramedics who use these electronic devices and guidelines daily are better prepared for the use of these technologies at a mass casualty incident. If smart glasses are developed further and become widely spread, augmented reality will become a superb opportunity for guideline-conforming operation with free usable hands. In addition to the presentation of guidelines, they can record relevant information by using an integrated camera or can be used as a tool for telemedical support.

### Limitations

A limiting factor for the presentation of guidelines with smart glasses is the low battery life of many models. Moreover, numerous models, including the model used in this study, are not suited for spectacle wearers. The operating concept of the used smart glasses with the optical touchpad and the switch is surely complex in the beginning, but easier user interfaces can be expected in the future. Another limitation of our study design is the adaptation to the guideline apart from the adaptation to the electronic devices. These parameters cannot be evaluated separately; therefore, the influence of the adaptation to the guideline on the speed of operation cannot be calculated. Through the preparation of comparable theoretical patient cases in 3 runs, it was possible to simulate and analyze the time and correctness of the triage. It certainly would have been more realistic to use, for example, a real disaster operation, though this would hardly be viable compared to a simulation. In this case, the examination of an alternative guideline that can be implemented into a real work environment can be planned.

Future studies should also focus on the age and experience of the test subjects, as this study is limited by test subjects’ average age of 24 years, which is certainly a young age and includes an already existing experience and familiarity with the developed technological devices. As medical staff consists of people of various ages and age generations, future studies should also evaluate the influence of the age generation on the usage of electronic devices for medical algorithm and decision-making. Further, experienced paramedics or other persons that become more familiar with the algorithm because of many triages might not strictly use a triage algorithm but use it only in a case of need. Therefore, future studies should analyze how often paramedics use a triage algorithm in reality and if the use of the triage algorithm in the course of many triages is reduced because they become more familiar with the triage (algorithm).

### Conclusion

In summary, the presentation of a guideline on a tablet as well as in the form of augmented reality achieved good results even with inexperienced users. The exemplary guideline was securely implemented. The implementation using smart glasses took more time owing to its more complex operating concept but could be accelerated in the course of the study after adaptation. The tablet, however, with its touchscreen as a familiar operating concept, achieved faster results. This operating concept has nevertheless the disadvantage of the use of both hands, which can be especially relevant in departments with special hygienic requirements or a high proportion of manual occupation. Especially in a non–time-critical working area, a guideline presentation with smart glasses can be implemented and a free-hand occupation can be executed. Further development of the operating concepts in the future or a highly intense training can speed up the processes, thereby making smart glasses a usable tool for guideline-conforming technical decision support.

## Abbreviations

AUDIME: Augmented Disaster Medicine
PRIOR: primary ranking for initial orientation in rescue service
